# Adaptive laboratory-evolved MRSA with PPEF manifests cross-susceptibility to oxacillin and hypersensitivity to ciprofloxacin

**DOI:** 10.1128/spectrum.02974-24

**Published:** 2025-07-15

**Authors:** Vikas Maurya, Raja Singh, Shruti Kutmutia, Bibha Chaudhary, Souvik Bhattacharjee, Vibha Tandon

**Affiliations:** 1 Special Centre for Molecular Medicine, Jawaharlal Nehru University 28754, New Delhi, India; 2 Department of Microbiology and Immunology, Emory University 1371, Atlanta, Georgia, USA; 3 Bacalt Biosciences Private Limited, Bengaluru, Karnataka, India; 4 Institute of Bioinformatics and Applied Biotechnology 29127, Bengaluru, India; 5 CSIR-Indian Institute of Chemical Biology 30156, Kolkata, West Bengal, India; Pontificia Universidade Catolica do Parana, Curitiba, Brazil

**Keywords:** antimicrobial resistance, PPEF, adaptive laboratory evolution, next-generation sequencing

## Abstract

Emerging resistance to current antibiotics is a global threat to human health. Therefore, comprehending the mechanism behind antibiotic resistance holds paramount importance. In the pursuit of finding new antibacterial agents, our group has developed a small molecule, PPEF (2′-(4-ethoxyphenyl)-5-(4-propylpiperazin-1-yl)-1H,1′H-2,5′-bibenzo(d)imidazole), having bisbenzimidazole as a pharmacophore, targeting bacterial type IA topoisomerase, a novel drug target in bacteria. We examined the emergence of mutations leading to PPEF resistance in laboratory-evolved *Staphylococcus aureus* strains. The growth curve revealed that *S. aureus* 25923 PPEF-resistant (SA-PR) and methicillin-resistant *S. aureus* 43300 PPEF-resistant (MRSA-PR) attained stationary phase earlier than their respective reference strains. RNA sequencing analysis revealed that *atpD* (ATP synthase gene) was downregulated by 2 log_2_-fold in both SA-PR and MRSA-PR strains, whereas there was 10 to 13 log_2_-fold downregulation of *mecR1* (methicillin resistance-inducing gene), *ble* (bleomycin resistance-inducing gene), *blaZ* (beta-lactamase), *pbp* (penicillin-binding protein gene), *ermA* (rRNA adenine methyltransferase gene), and *kdpB* (potassium-transporting ATPase) in the MRSA-PR strain. Quantitative reverse-transcriptase PCR data confirmed these results. Additionally, MRSA-PR showed a 5 log_2_-fold upregulation of *comG* and a 9 log_2_-fold downregulation of *topB*, indicating increased genomic variability and stress adaptation contributing to resistance. Genomic sequencing revealed deletions of resistance genes, including *aac(6')-aph(2'')*, *aadD*, *mecA*, and *blaZ* in MRSA-PR, resulting in a gain in resistance and a diminishing returns epistasis pattern in PPEF-evolved *S. aureus* strains. This led to the development of an evolved MRSA-PR strain susceptible to oxacillin, ciprofloxacin, gentamicin, and imipenem. Our findings indicate that adaptation to PPEF has increased antibiotic susceptibility, thereby changing the clinical outcomes of infections.

**IMPORTANCE:**

This study investigates how *Staphylococcus aureus* bacteria, including methicillin-resistant *Staphylococcus aureus* (MRSA) strain, develop resistance to a new candidate antibacterial compound, PPEF (2′-(4-ethoxyphenyl)-5-(4-propylpiperazin-1-yl)-1H,1′H-2,5′-bibenzo(d)imidazole). The research found that resistant strains grew slower and showed significant changes in the activity of genes related to antibiotic resistance. Some resistance genes were deleted in the resistant MRSA strain, making it more sensitive to other antibiotics like oxacillin and ciprofloxacin. These findings highlight how resistance to PPEF leads to increased sensitivity to conventional antibiotics. This suggests that developing combination therapies of PPEF with other antibiotics could optimize treatment regimens and slow resistance evolution. This study also indicates that the antibiotic regimens could be designed to force resistant bacteria into evolutionary trade-offs, where they lose resistance to widely used antibiotics while gaining resistance to a new compound like PPEF.

## INTRODUCTION

Human health is under tremendous threat due to the emergence of multidrug-resistant pathogenic bacteria (1, 2). Antimicrobial resistance-associated deaths were approximately 5,000,000 in 2021 and are speculated to increase up to 10 million by 2050, as estimated by the World Health Organization (WHO) (3). *Staphylococcus aureus* is a highly adaptable and versatile bacterium that asymptomatically colonizes approximately one-third of the global population, primarily on the skin and nasopharyngeal membranes (1). It is capable of causing a wide range of diseases, including bacteremia, septicemia, urinary tract infections, osteomyelitis, otitis, endocarditis, and mastitis (4). The bacterium exhibits remarkable metabolic versatility and pharmacological resistance, allowing it to thrive in diverse environments (5). *S. aureus* employs multiple resistance mechanisms to evade antibiotic action. These include the production of β-lactamase enzymes to neutralize β-lactam antibiotics, efflux pumps to expel antibiotics like tetracyclines, reduced accumulation of macrolides, synthesis of aminoglycoside-modifying enzymes to deactivate aminoglycoside antibiotics, modification of DNA gyrase and topoisomerase IV to resist quinolones, and activation of *mec* genes, which alter penicillin-binding proteins (6). Methicillin-resistant *Staphylococcus aureus* (MRSA) is a particularly concerning strain of *S. aureus* and has been recognized by the WHO as one of the leading causes of hospital-acquired infections. MRSA poses a significant clinical threat, contributing to persistently high morbidity and mortality rates. Due to its extensive antibiotic resistance, MRSA remains a major contributor to healthcare-associated infections and serious conditions such as bacteremia, septicemia, and osteomyelitis (1, 3).

Resistance to an extensive list of β-lactams through the acquisition of the transposable element *mec* (*SSCmec*) is designated as MRSA (7). Penicillin-binding protein 2a (PBP2a) is encoded by the *mecA* gene, which has lower affinity for β-lactams and performs crosslinks with bacterial peptidoglycan in the presence of antibiotics (7, 8). MRSA with complicated evolution has acquired resistance to different antibiotics from penicillin/methicillin to quinolone and vancomycin (9). *Enterococcus faecalis* has the *vanA* operon in the plasmid-borne transposon Tn1546, which transfers the *vanA* gene cluster to *S. aureus. vanA* modifies or eliminates the vancomycin-binding site, leading to the development of vancomycin-resistant *S. aureus* (VRSA) (9).

Understanding the mechanism of resistance to antibiotics is essential for discovering novel antimicrobial drugs, as well as in the control of resistant pathogens in healthcare centers (2). Through a phenomenon called collateral sensitivity, the increasing resistance to one antibiotic is often associated with increased susceptibility to a second antimicrobial agent. According to an *in vitro* study, 74% of laboratory‐evolved strains resistant to certain antibiotics have shown collateral sensitivity to one or more antimicrobial agents (10).

The rapid emergence of multi-antimicrobial-resistant strains has created an urgent need for the development of novel therapeutic agents by identifying new potential antimicrobial targets or discovering new chemical entities as antimicrobial agents (8, 11). Previously, we synthesized a series of bisbenzimidazole derivatives in our laboratory with different piperazines at one end and aromatic aldehydes with varying substituent on the phenyl ring at the other. We earlier reported that 2′-(4-ethoxyphenyl)-5-(4-propylpiperazin-1-yl)-1H,1′H-2,5′-bibenzo(d)imidazole (PPEF), a bisbenzimidazole, preferentially targets type IA topoisomerase over gyrase and human topoisomerase I (12–14). PPEF was identified as a potent type IA topoisomerase I inhibitor, exhibiting a half-maximal inhibitory concentration (IC50) of 10 µM (15). This compound affects the regulation of DNA, leading to accumulation of cleaved DNA, and thus acts as a poison inhibitor (14). It also showed potent activity against methicillin- and vancomycin-resistant *S. aureus* (16, 17).

In this study, we tested the hypothesis that the adaptive modulation in MRSA upon PPEF treatment can cause widespread gene expression changes which, in turn, alter cellular phenotypes, including altered susceptibilities to other antimicrobial agents. A correlation was also established between the changes in the mutational landscape and differential expression of genes in PPEF-evolved resistant *S. aureus*. It was noted that in MRSA, the expression of genes involved in carotenoid biosynthetic process and ATP synthesis was downregulated, whereas the arginine deaminase pathway was upregulated. Whole-genome sequencing was used to characterize the molecular mechanisms involved in resistance and collateral responses. This study sought to determine the resistance and cross-resistance evolution of *S. aureus* against PPEF and to decipher the resistance mechanisms against PPEF.

## RESULTS AND DISCUSSION

### *S. aureus* with distinct phenotypes evolved from serial PPEF treatment

**Fig 1:**
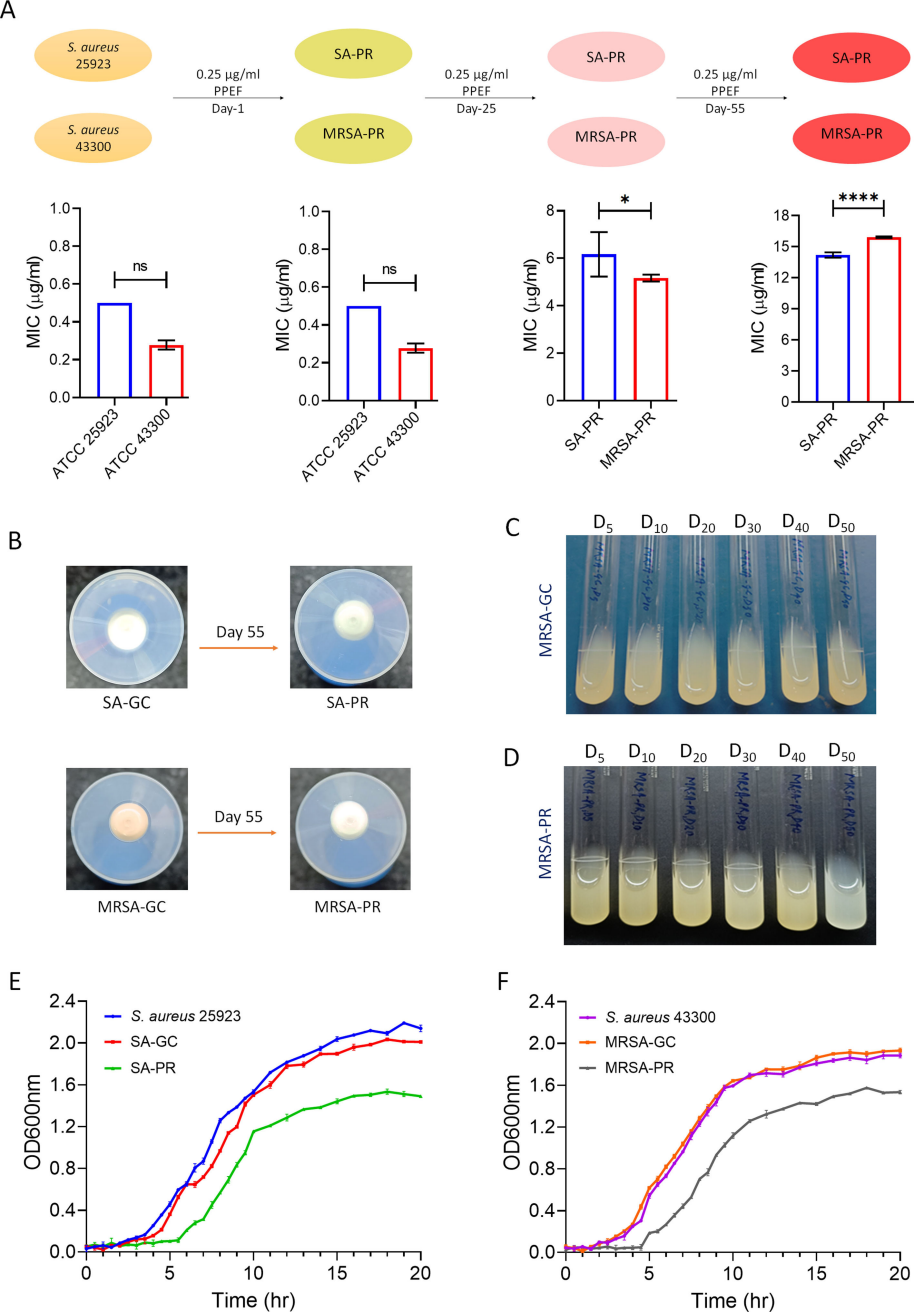
*S. aureus* strains exhibit adaptive evolution in response to escalating levels of PPEF, accompanied by phenotypic alterations. (**A**) *S. aureus* was exposed to PPEF for 55 days to attain PPEF resistance (PR). The figure represents data from three separate experiments (*P < 0.01, ****P < 0.0001, and ns, not significant, were calculated using two-way analysis of variance). (**B**) Reduction in pigmentation observed in the evolved strains of MRSA-PR pelleted at the bottom of a 50 mL tube. (**C, D**) A visual assessment of the reduction in pigmentation in the evolved strains of MRSA-PR for 50 days (**D**) in comparison to MRSA-GC. (**E, F**) Growth curve of the evolved strains SA-GC, SA-PR, MRSA-GC, and MRSA-PR relative to *S. aureus* ATCC 25923 and MRSA 43300, respectively. The figure represents data from three separate experiments.

We followed the evolution of *S. aureus* ATCC 25923 (*S. aureus* growth control [SA-GC]) and ATCC 43300 (MRSA-GC) under PPEF treatment regimens. The timeline for the evolution experiment is shown in Fig. 1A, wherein the bacterial cultures were serially treated with PPEF. We observed that the PPEF-sensitive strains ATCC 25923 (SA-GC) and ATCC 43300 (MRSA-GC) switched to PPEF-resistant phenotype (*S. aureus* 25923 PPEF-resistant [SA-PR] and methicillin-resistant *S. aureus* 43300 PPEF-resistant [MRSA-PR]). SA-PR and MRSA-PR reached minimum inhibitory concentration (MIC) values of 14 and 16 µg/mL, respectively, following 55 days of PPEF treatment (Fig. 1A). Further increases in MIC values were not checked (Fig. 1A). After 55 days serial exposure to PPEF, the MIC values of obtained SA-PR were 28 times higher than that of the ancestral strain and 32 times higher for MRSA-PR.

Interestingly, *S. aureus* strains did not revert to PPEF-sensitive phenotype after being cultured in a PPEF-free medium for 10 days (MIC remained 28–32 times higher than the ancestral strain). Gram staining and 16S rRNA sequencing results proved that there was no contamination during the PPEF serial treatment process (see Fig. S1 at https://github.com/Vibhatandon1860/Microbiology-Spectrum).

### Progressive PPEF exposure induces clinical sensitivity to multiple classes of antibiotics

The MIC of PPEF in the sensitive strains SA-GC and MRSA-GC and the resistant strains SA-PR and MRSA-PR was determined by the broth microdilution assay. We observed changes in the MIC values for several classes of antibiotics in both the strains (Table 1). The concentration of antibiotics at which no growth was observed, defined as the MIC breakpoint, was determined for vancomycin, ciprofloxacin (CIP), kanamycin, gentamicin, co-trimoxazole, colistin, tetracycline, chloramphenicol, and ofloxacin, and five different beta-lactam antibiotics (ampicillin, oxacillin, cefoxitin, cefotaxime, and imipenem). The β-lactam drugs were chosen as representative of different generations of cephalosporin antibiotics and a carbapenem, respectively. Clinical resistance was determined using CLSI breakpoints (18).

**TABLE 1:** Antibiotic susceptibility profile of *S*. *aureus* SA-GC and MRSA-GC and *S*. *aureus* SA-PR and MRSA-PR

Antibiotic	*S. aureus* (µg/mL)		MRSA (µg/mL)	
	SA-GC	SA-PR	MRSA-GC	MRSA-PR
Vancomycin	2.0 ± 0.09	1.0 ± 0.02	0.25 ± 0.001	4.0 ± 0.09
Ciprofloxacin	0.25 ± 0.03	0.25 ± 0.01	>64 ± 0.04	0.25 ± 0.01
Oxacillin	0.125 ± 0.1	0.25 ± 0.07	>128 ± 0.12	0.25 ± 0.03
Cefotaxime	16 ± 0.05	1.0 ± 0.06	>128 ± 0.05	1.0 ± 0.02
Ampicillin	0.25 ± 0.05	0.25 ± 0.04	>128 ± 0.14	0.25 ± 0.01
Kanamycin	1 ± 0.03	1.0 ± 0.001	>128 ± 0.02	2.0 ± 0.08
Cefoxitin	32 ± 0.01	1.0 ± 0.15	>128 ± 0.31	2.0 ± 0.01
Gentamicin	0.5 ± 0.04	0.5 ± 0.01	>128 ± 0.07	32 ± 0.03
Co-trimoxazole	16 ± 0.03	1.0 ± 0.01	>128 ± 0.03	1.0 ± 0.001
Imipenem	0.25 ± 0.04	>128 ± 0.05	>128 ± 0.012	>128 ± 0.2
Colistin	0.5 ± 0.06	8.0 ± 0.10	>128 ± 0.03	128 ± 0.02
Tetracycline	0.25 ± 0.01	>256 ± 0.03	0.25 ± 0.04	1 ± 0.03
Chloramphenicol	8.0 ± 0.01	4.0 ± 0.01	8 ± 0.05	4.0 ± 0.01
Ofloxacin	0.25 ± 0.06	0.25 ± 0.06	>128 ± 0.03	0.25 ± 0.06

The MIC of SA-PR and MRSA-PR did not remain the same as the ancestral strain (SA-GC and MRSA-GC). Serial exposure to sub-MICs can exert favorable selective pressure, leading to the emergence of resistant phenotypes, consistent with prior reports (19, 20). The treatment of PPEF affected the resistance profile of known or standard antibiotics. We also observed that the treatment of PPEF made the evolved strains more sensitive toward standard antibiotics (Table 1). The SA-PR cells became resistant to tetracycline and imipenem (>128 µg/mL), which was sensitive against the SA-GC strain. MRSA-GC was resistant to ciprofloxacin, oxacillin, cefotaxime, ampicillin, kanamycin, cefoxitin, gentamicin, co-trimoxazole, imipenem, colistin, and ofloxacin. However, with the progressive PPEF exposure, MRSA-PR became sensitive to them. The results demonstrate that the gain in resistance against PPEF causes sensitivity to other antibiotics. This phenomenon aligns with the well-documented development of cross-resistance in gram-positive bacteria, such as *S. aureus* (21) and *Mycobacterium tuberculosis* (22), when exposed to ciprofloxacin. This finding suggests that bacterial adaptation to one antibiotic may reduce resistance to others. However, it is not well established how these trade-offs between decreased virulence and increased sensitivity could be utilized for manipulating antibiotic resistance as the underlying molecular mechanism. A deeper exploration is critical to deduce the reason for the sensitivity of MRSA to another antibiotic.

### Epistatic interactions underlie evolvability alterations

Characterization of colony morphology often complements conventional microbial identification in the detection of intra-strain diversity (23). *S. aureus* produces the yellow pigment staphyloxanthin (STX) synthesized by genes in the crtOPQMN operon, and characteristic gold-colored colonies are formed on all rich media including tryptic soy agar at 37°C, brain heart infusion agar, and Luria-Bertani agar (24). The STX helps bacteria defend against reactive oxygen species. We observed no change in the colony color for SA-GC after progressive PPEF exposure (Fig. 1B). In contrast, the colony MRSA was initially dark yellow in color, which changed to pale yellow on progressive exposure to PPEF (Fig. 1B). It has been previously reported that *CspA* regulates pigment production in *S. aureus* through a *SigB*-dependent mechanism (25). Thus, the *cspA*-negative mutant of methicillin-resistant *S. aureus* strain COL is devoid of the ability to produce pigment. The change in colony color of MRSA-PR was further confirmed in liquid culture of the evolved strains at days 0, 5, 10, 20, 30, 40, and 50 as shown in Fig. 1C and D. The growth curves (optical density at 600 nm [OD_600_] as a function of time) were plotted for the evolved strains SA-GC and SA-PR and MRSA-GC and MRSA-PR relative to *S. aureus* 25923 and MRSA 43300, respectively. The curve represented the lag phase, exponential growth, followed by a stationary phase of saturation. It was observed that saturation for the PPEF evolved strains (SA-PR and MRSA-PR) was reached earlier than that for the standard strain of *S. aureus* (Fig. 1E and F). The doubling time was calculated for SA-PR, MRSA-PR, SA-GC, and MRSA-GC using GrowthRates 6.2.1. It was observed that there was a change in the doubling time for the SA-PR and MRSA-PR (~42.2 and ~42.4 min) as compared to the standard SA-GC and MRSA-GC (~35.0 and ~36.3 min). PPEF resistance resulted in a 0.8-fold increase in doubling time for SA-PR and MRSA-PR, indicating an impact on cell growth and cell cycle, with resistant strains reaching saturation earlier than reference strains.

### Differentially expressed genes (DEGs) in *S. aureus* SA-PR and MRSA-PR strains

To further identify global gene signatures intrinsic to the PPEF resistance, we performed DEG analysis by comparing the mean expression values for all genes across equivalent clusters. The computational analysis was performed for the DEGs obtained in *S. aureus* in the presence or absence of PPEF to deduce the changes in gene expression after exposure to PPEF. The quality (Q30) of the raw reads was 93.59, 92.06, 92.23, and 93.28% for SA-GC, SA-PR, MRSA-GC, and MRSA-PR, respectively (Table S2; also Fig. S2 and S3 at https://github.com/Vibhatandon1860/Microbiology-Spectrum). A uniform distribution of the read counts was observed for SA-GC, SA-PR, MRSA-GC, and MRSA-PR, followed by an absence of the multimodal pattern, which suggests homogeneity within the replicate samples (see Fig. S4 at https://github.com/Vibhatandon1860/Microbiology-Spectrum). Principal component analysis showed clustering for SA-GC versus SA-PR and MRSA-GC versus MRSA-PR with a principal component (PC1) variance of 59% and 95% and PC2 variance of 26% and 4% for SA and MRSA, respectively (see Fig. S5 at https://github.com/Vibhatandon1860/Microbiology-Spectrum). We observed a high number of data points falling above the one threshold and more below −1 on the *y*-axis in the MA plot, indicating that a significant number of genes were being upregulated and downregulated in MRSA-PR compared to SA (see Fig. S6 at https://github.com/Vibhatandon1860/Microbiology-Spectrum).

**Fig 2:**
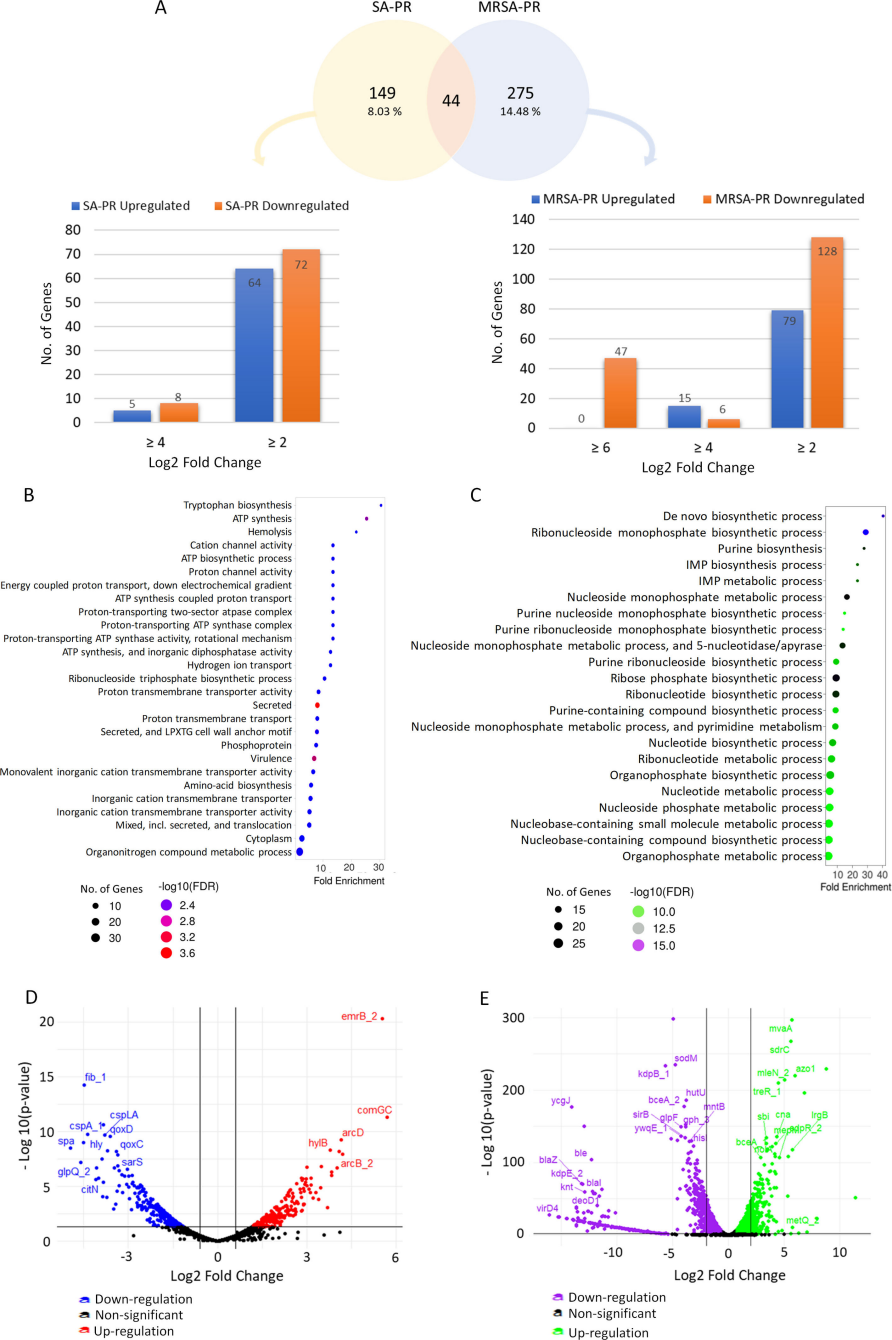
Genes exhibiting differential regulation in *S. aureus* strains challenged with PPEF, as determined by transcriptional gene expression analysis using next-generation sequencing. (**A**) Differential gene expression profile of SA-PR and MRSA-PR as compared to their respective reference strains. (**B, C**) Enriched molecular pathways in PPEF-evolved *S. aureus* strains in SA-PR and MRSA-PR, respectively. (**D**) Volcano plot depicting difference between transcriptomics data of SA-PR and SA-GC samples. (**E**) Plotted points show average difference in gene expression between MRSA-PR and MRSA-GC. Each point represents a gene in the SA and MRSA transcriptomes. The log_2_-fold change indicates the mean expression level for each gene at the *x*-axis. The *y*-axis represents an adjusted *P*-value of genes. Genes with log_2_-fold changes having cutoff ≥2 and –log_10_ (*P*-value) cutoff ≥2.0 were considered upregulated and grouped on the right. Genes with log_2_-fold changes less than or equal to −2 and –log_10_ (*P*-value) ≥2.0 were considered downregulated and grouped on the left. Black dots indicate non-significant genes.

To explore the molecular mechanisms of PPEF resistance, the transcriptomic profile of SA-PR and MRSA-PR was mapped to SA-GC and MRSA-GC, respectively. The transcriptome analysis of SA-PR showed a total of 1,854 genes to be differentially expressed, in which 69 were upregulated and 80 were downregulated with a cutoff of log_2_-fold change ≥2 and *P*-value <0.01 (Fig. 2A). The transcriptome analysis on the MRSA-PR depicted that out of 1,898 DEGs, 94 genes were upregulated, and 181 genes were downregulated with log_2_-fold change ≥2 with a *P*-value <0.01 (Fig. 2A).

The gene ontology (GO) enrichment analysis revealed that the differentially expressed genes were primarily involved in various biosynthetic processes. In SA-PR, the genes were mainly enriched in tryptophan biosynthesis, ATP synthesis, and virulence (Fig. 2B). Conversely, in MRSA-PR, the genes were enriched in purine biosynthesis, ribonucleotide synthesis, and the IMP biosynthesis process (Fig. 2C). These comprehensive changes in gene expression across the genome could be observed by volcano plot (Fig. 2D and E).

The gene expression landscape was further established encompassing the top 25% regulated genes in SA-PR and MRSA-PR. It was observed that ComGC operon protein 3 and multidrug export protein EmrB were upregulated 5 log_2_-fold in SA-PR cells. The *comG* operon, important for assembling type IV transformation pili, is required for DNA uptake in *S. pneumoniae* and *Bacillus subtilis* and includes *comGC* and three additional small open reading frames (*comGD, comGE,* and *comGF*) (26, 27). Ornithine carbamoyl transferase (*arcB_2*), catabolic arginine/ornithine antiporter (*arcD*), and bacitracin export ATP-binding protein (*bceA*) genes were upregulated 3 log_2_-fold in SA-PR (see Fig. S7A at https://github.com/Vibhatandon1860/Microbiology-Spectrum). Conversely, in MRSA-PR, six genes (*azo1, metQ_2, lrgB, mvaA, sdrC, mleN_2*) were upregulated with a 5 log_2_-fold change, and eight different genes (*cna, cobQ, mepM, glnQ, norB_3, murB, czcD_1, entA_5*) were upregulated with a 4 log_2_-fold change (see Fig. S7B). *Azo* is involved in xenobiotic metabolism, while *MetQ* is a binding protein for L- and D-methionine (28). Additionally, the *mvaA* gene, essential for peptidoglycan synthesis in *S. aureus*, and *sdrC* are linked to the production of virulence factors that facilitate biofilm formation (29).

However, more than seven different genes (*spa, glpQ_2, fib_1, cspA_1, fadA, ywqD_1, znuC_2*) were downregulated with a 4 log_2_-fold change, and 14 different genes (*cap8A_1, citN, sbi, cspLA, nuc, hly, qoxD, map_1, ywqE_1, qoxC, sarS, hel, glpK*) were downregulated 3 log_2_-fold in SA-PR (see Fig. S7C). In MRSA-PR, 46 different genes exhibited a downregulation between −16 and −5 log_2_-fold change, mainly induced by PPEF treatment (see Fig. S7D and E). The most significantly downregulated genes included *kdpABCDE* (potassium-transporting ATPase), *mecI, mecR1* (methicillin regulatory and resistance), *ant1* (streptomycin 3''-adenylyltransferase), *appA* (oligopeptide-binding protein), *blaI* (penicillinase repressor), *blaR1* (regulatory protein), *blaZ* (beta-lactamase), and *ble* (bleomycin resistance protein). No significant changes were observed at the transcriptomics level in type I topoisomerase genes (*topA* and *topB*) in SA-PR. However, in MRSA-PR, the *topA* and *topB* genes were downregulated by 1.17- and 9.79-fold, respectively.

### Homogeneity in differential expression of genes contributes to adaptive PPEF resistance in SA-PR and MRSA-PR

**Fig 3:**
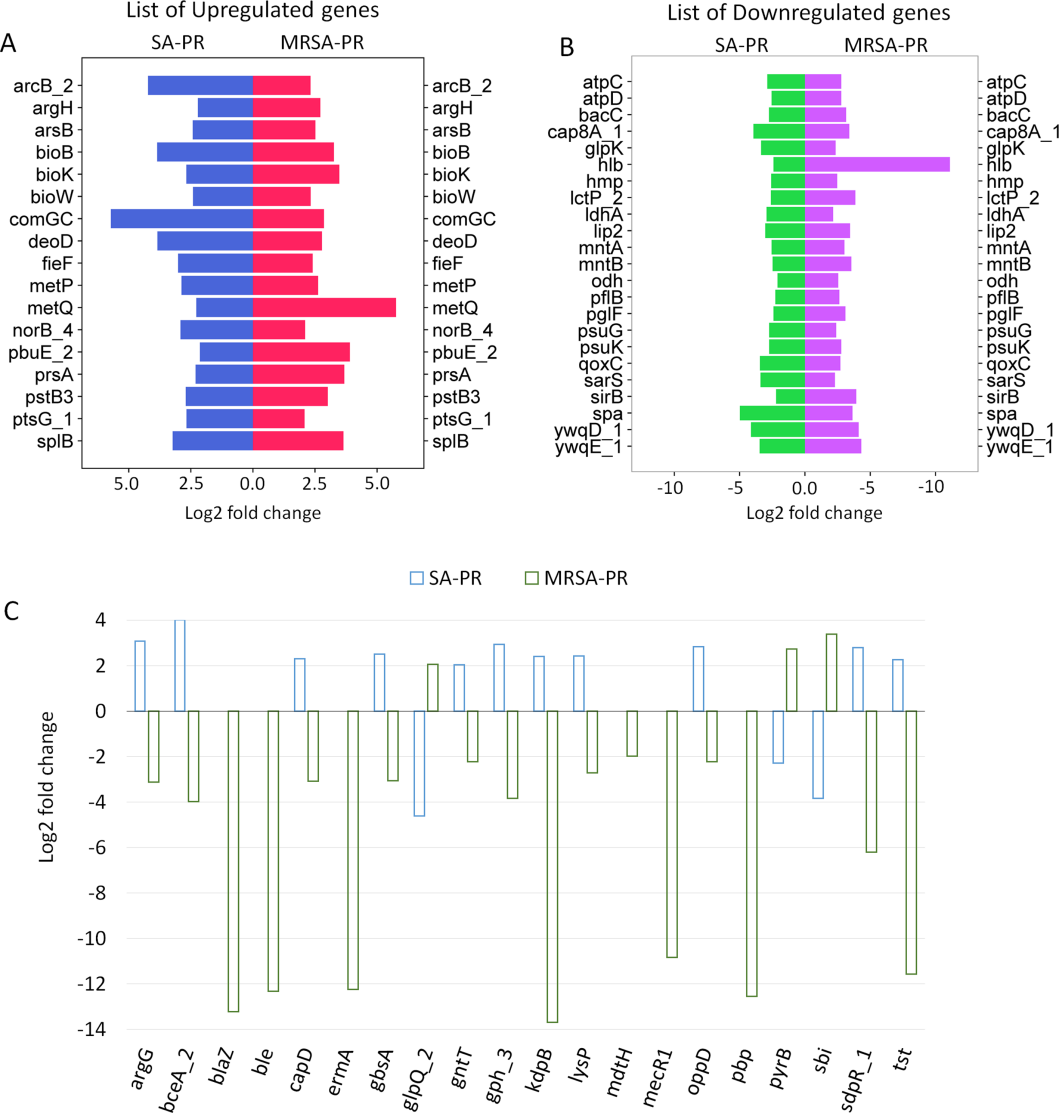
Similar gene expression profile of SA-PR and MRSA-PR. (**A**) List of similar genes which are upregulated in both SA-PR and MRSA-PR. (**B**) List of similar genes which are downregulated in both SA-PR and MRSA-PR. (**C**) List of genes expressed in SA-PR and MRSA-PR responsible for the development of resistance to PPEF and sensitivity to known antibiotics.

The transcriptomic profiles of treated SA and MRSA revealed differential patterns of gene expression involved in the development of resistance to PPEF and sensitivity of the PR strains to the standard antibiotics. The upregulation of genes such as *argH, comGC, metP, metQ,* and *norB*, combined with the downregulation of genes like *atpC, atpD, sarS, spa, mntA*, and *mntB*, contributed to the resistance of SA-PR and MRSA-PR strains to PPEF treatment (Fig. 3A and B). The upregulation of argH may increase arginine levels, which can facilitate biofilm formation and enhance antibiotic resistance. Conversely, the downregulation of ATP synthase subunits can decrease ATP production, potentially inducing a state of low metabolic activity in *S. aureus* and MRSA. This low-energy state may alter membrane potential and limit antibiotic uptake, thereby contributing to antibiotic resistance. Moreover, ATP synthase inactivation has been linked to heightened susceptibility to polymyxins, gentamicin, and nitric oxide (30). Additionally, mntA and mntB, components of the manganese uptake system, play a crucial role in oxidative stress protection. Their downregulation compromises this defense mechanism, increasing bacterial vulnerability to oxidative damage and quinolones (31).

It was also observed that the *argG, bceA, capD*, and *gbsA* genes were downregulated in MRSA-PR (Fig. 3C). The downregulation of the *argG* gene, involved in arginine biosynthesis, increased MRSA-PR’s sensitivity to aminoglycosides, likely due to metabolic changes (32). The *bceA* gene, part of the *bceAB* operon encoding components of an ATP-binding cassette (ABC) transporter system, was also downregulated. This led to MRSA-PR’s increased sensitivity to bacitracin and vancomycin (33). The *capD* gene, which plays a role in the biosynthesis of capsular polysaccharides and is part of the capsule synthesis pathway, affects bacterial virulence and resistance. Its downregulation altered MRSA-PR’s susceptibility to penicillin and cephalosporins (34). The *kdpB* gene, part of the *kdp* operon encoding the Kdp ATPase system responsible for potassium uptake, had altered expression in MRSA-PR, impacting its susceptibility to gentamicin and kanamycin (35). The *lysP* gene, which encodes a lysine transporter involved in lysine uptake, was downregulated, affecting MRSA-PR’s sensitivity to penicillin, cephalosporins, and colistin (36). In contrast, the *glpQ, pyrB,* and *sbi* genes were downregulated in SA-PR (Fig. 3C). The *glpQ* gene, encoding glycerol-3-phosphate phosphatase, is involved in glycerol-3-phosphate metabolism and plays a role in phosphate regulation and energy metabolism. The altered function of *glpQ* in SA-PR affected its sensitivity to penicillin and cephalosporins, influenced by changes in metabolic pathways and cellular stress responses. The *pyrB* gene, encoding a component of the aspartate transcarbamoylase enzyme involved in pyrimidine biosynthesis, was downregulated, resulting in increased sensitivity to rifampin, ciprofloxacin, and levofloxacin. The downregulation of the *sbi* gene impacted SA-PR’s susceptibility to methicillin and penicillin (37).

We observed that the genes *blaz, ble, mdtH, mecR1*, and *pbp* were exclusively downregulated in MRSA-PR, while these genes were not present in SA-PR. The *blaZ* gene encodes beta-lactamase, which provides resistance to penicillin, cephalosporins, monobactams, and carbapenems. The decreased expression of blaZ led to increased sensitivity of MRSA-PR to penicillin. The *ble* gene encodes a protein that inactivates bleomycin; its downregulation resulted in increased sensitivity to bleomycin and other antibiotics such as tetracycline and chloramphenicol due to reduced expression of the multidrug efflux pump gene *mdtH* (38). Additionally, the downregulation of *mecR1*, which is part of the *mec* operon that includes the *mecA* gene encoding PBP2a, increased the strain’s sensitivity to methicillin.

### Network analysis of the DEGs and identification of clusters

**Fig 4:**
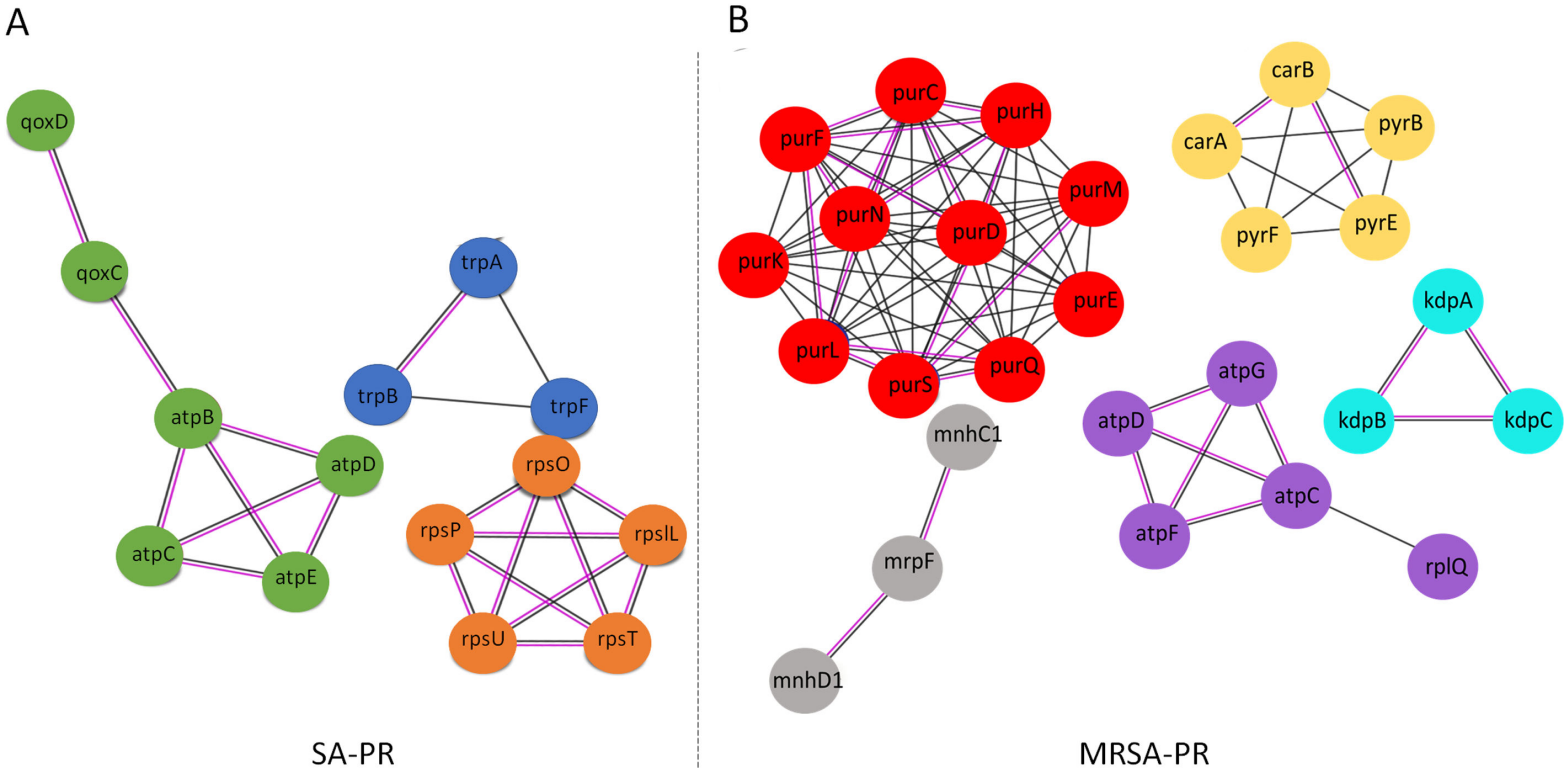
Protein-protein interaction networks for SA-PR (**A**) and MRSA-PR (**B**) were analyzed using Markov cluster algorithm (MCL) clustering with an inflation parameter of 3 and an interaction score threshold of 0.9. The clusters are color-coded for SA-PR. Cluster 1 is represented by orange bubbles, cluster 2 by green, cluster 3 by blue, and for MRSA-PR, cluster 1 is represented by red bubbles, cluster 2 by yellow, cluster 3 by violet, cluster 4 by sky blue, and cluster 5 by gray.

To investigate the correlation between the differential expression of genes during the treatment of PPEF, we used the STRING database (39) to identify potential interactions. A protein-protein interaction (PPI) network between DEGs was constructed for the PPEF-treated MRSA with the highest confidence of 0.9 and high false discovery rate (FDR) stringency (1%). Six significant clusters of highly interconnected nodes were identified by k_means_ clustering. MRSA-PR cluster 1 consisted of genes, namely *purF, purC, purH, purM, purE, purS, purD,* and *purN,* that were related to the *de novo* purine biosynthesis pathway that involves a series of pathways responsible for the synthesis and degradation of purine nucleotides. Cluster 2 included *carA, carB, pyrB, pyre,* and *pyrF* genes that play a role in the biosynthesis of pyrimidine nucleotide. *atpD, atpG, atpC, atpF,* and *rplQ* genes were evident in cluster 3. These genes are involved in bacterial ATP synthesis, and ribosomal function was related to ATP synthesis. *kdpA, kdpB,* and *kdpC* genes were found in cluster 4. These genes are involved in high-affinity potassium transport. These genes are essential for potassium regulation, which is crucial for maintaining cell turgor and normal homeostasis, and the *KdpD/KdpB* two-component system regulates this high-affinity potassium pump. The downregulation of *kdpD* and *kdpB* affects the regulation of virulence genes and mediates stress resistance (40). *mnhC1, mnhD1,* and *mnhF1* genes are components of the MnH complex. This complex is involved in the regulation of pH homeostasis and sodium ion transport. These genes formed cluster 5 and were downregulated in MRSA-PR. We could observe three significant clusters for evolved *S. aureus* (SA). Out of the three, there was one common cluster of genes involved in ATP synthesis in the PPI network constructed for SA (cluster 2) and MRSA (cluster 3) using the STRING database. The *rpIl*, *rpsO*, *rpsT*, and *rpsU* genes were found in cluster 1 involved in protein synthesis by contributing to to assembly, stability, and proper functioning of ribosomal subunit. Cluster 3 was involved in the tryptophan biosynthesis pathway (Fig. 4).

### Whole-genome sequencing reveals distinctive genetic changes because of progressive PPEF exposure

#### Genotypic characterization

**Fig 5:**
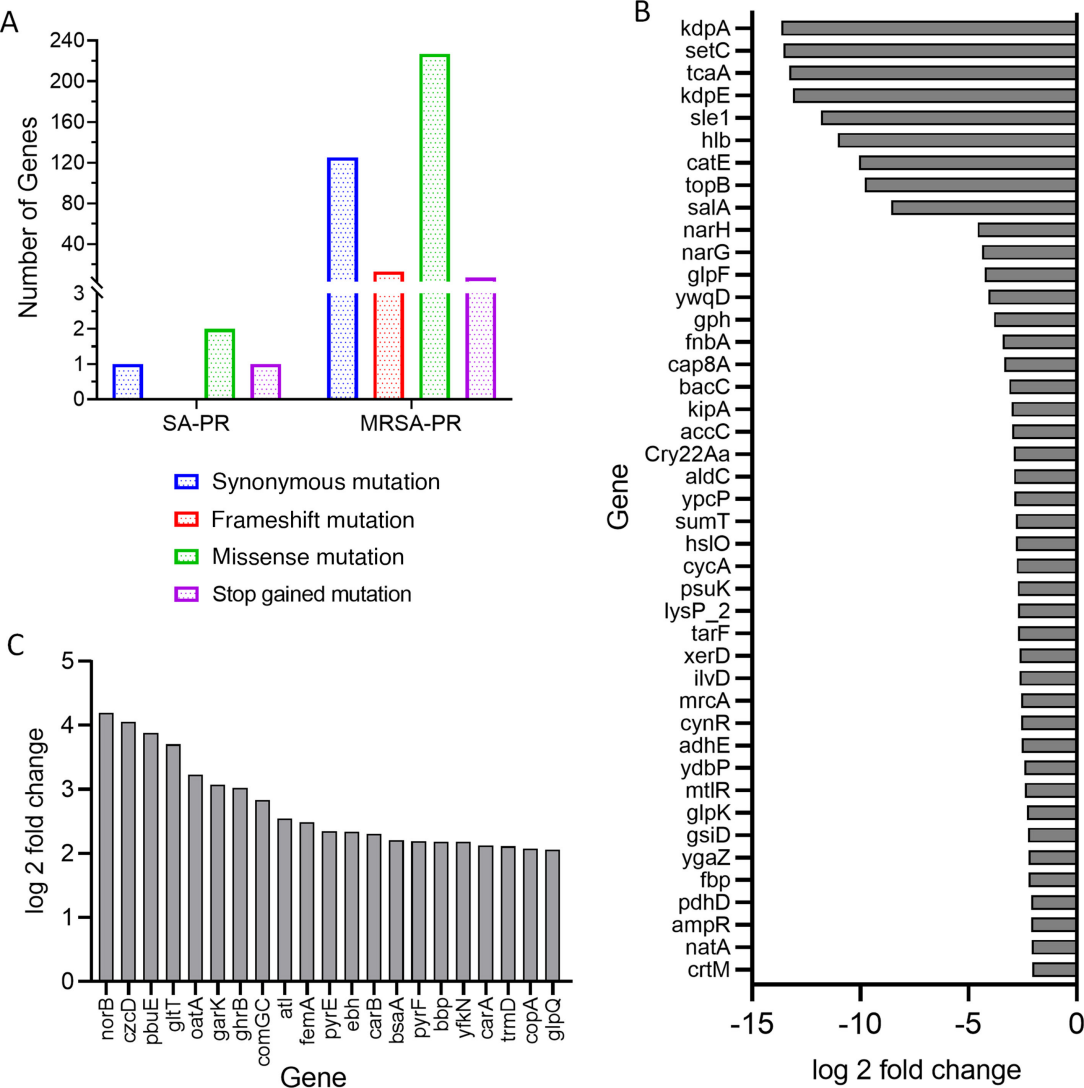
(**A**) Bar diagram depicting the number of genes associated with different types of mutation in SA-PR and MRSA-PR. (**B**) Expression profile (downregulation) of the genes involved in non-synonymous, frameshift, missense, and stop-gain mutation along with their log_2_-fold change. (C) Expression profile (upregulation) of the genes involved in non-synonymous, frameshift, missense, and stop-gain mutation along with their log_2_-fold change.

Genome sequences of SA-GC, SA-PR, MRSA-GC, and MRSA-PR were obtained through whole genome sequencing (WGS). The quality (Q30) of the raw reads was 92.88%, 92.76%, 90.44%, and 92.3% for SA-GC, SA-PR, MRSA-GC, and MRSA-PR, respectively (Table S3; also see Fig. S2 at https://github.com/Vibhatandon1860/Microbiology-Spectrum). We observed that SA-PR harbored non-synonymous substitutions in two genes, namely DNA-directed RNA polymerase (*rpoB*) and RNA polymerase sigma-B factor (*sigB*) in SA-PR (Fig. 5A).

In MRSA-PR, there were a total of 339 single nucleotide polymorphisms (SNPs) in which 201 were non-synonymous, 13 were frameshift mutations, and 7 were stop mutations (Fig. 5A). There were 13 frameshift mutations in *acuC*, *queG*, *argJ*, *alkA*, *alkA*, *sspA*, *hylB*, *leuA*, *xerD*, *pyrC*, *carA*, *bbp*, and *bsaA* and seven stop mutations in *recN*, *femA*, *asp*, *atl*, *gatD*, *gltA*, and *ghrB* genes of MRSA-PR with respect to the reference MRSA, as shown in Fig. 5B. Out of the 13 genes with frameshift mutations, three genes, *carA, bbp*, and *bsaA*, were upregulated. Additionally, the *femA* and *atl* genes were upregulated with stop-gain mutations. *FemA* gene is an essential factor for the level of methicillin resistance and plays a significant role in the formation of cell wall peptidoglycan (41). The acquisition of a stop-gain mutation in *femA* resulted in the appearance of a premature stop codon, leading to the production of truncated FemA protein. This led to the loss of methicillin resistance.

Out of 201 SNPs, 76 genes were downregulated, and 19 genes, namely *kdpA* (−13.64), *setC* (−13.54), *tcaA* (−13.28), *kdpE* (−13.11), *hlb* (−11.04), *glpQ* (−10.83), *catE* (−10.06), *topB* (−9.79), *salA* (−8.57), *narG* (−4.57), *narH* (−4.57), *glpF* (−4.24), *ywqD* (−4.07), *gph* (−3.82), *fnbA* (−3.43), *cap8A* (−3.35), *bacC* (−3.10), *kipA* (−3.01), were found to be downregulated with less than −3 log_2_-fold change. Fifty SNPs were upregulated, and the following genes exhibited expression levels greater than 3 log_2_-fold: *norB* (quinolone resistance protein), *czcD* [cadmium, cobalt, and zinc/H(+)-K(+) antiporter], *pbuE* (purine efflux pump), *oatA* (O-acetyltransferase), *gltT* (proton/sodium-glutamate symport protein), *garK* (glycerate 2-kinase), and *ghrB* (glyoxylate/hydroxypyruvate reductase B). Other important genes, namely extracellular matrix-binding protein (*ebh*), purine efflux pump (*pbuE*), DNA polymerase III subunit gamma (*dnaX*), translational initiation factor (*infB*), methionine import ATP-binding protein (*metN*_2), staphylococcal secretory antigen (*ssaA*), and multidrug efflux pump (*sdrM*), were upregulated, which helps in the development of resistance to PPEF.

### Modulation of virulence and biofilm formation in SA-PR and MRSA-PR

**Fig 6:**
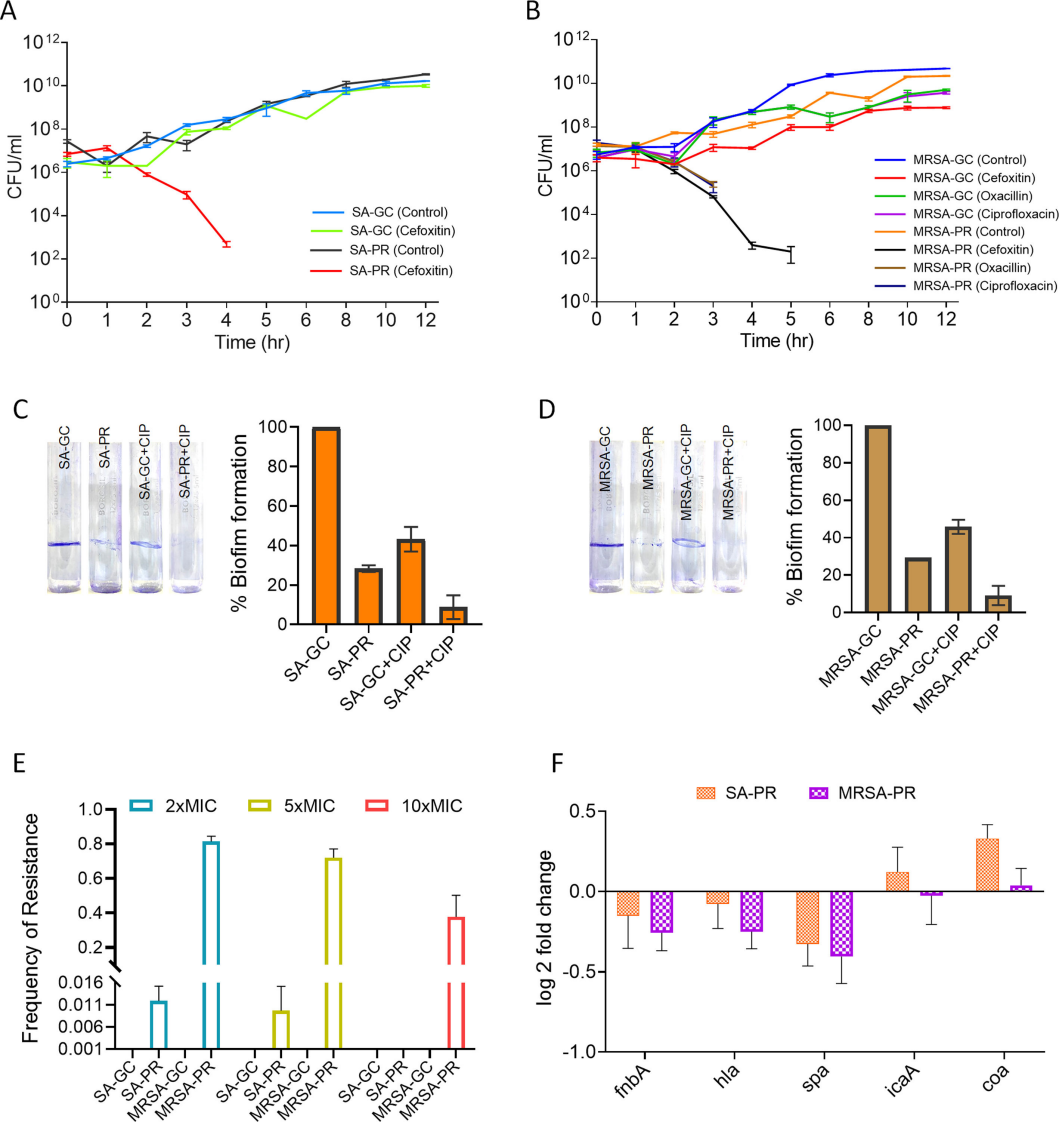
Effect of ciprofloxacin on virulence gene expression and biofilm formation in SA-PR and MRSA-PR. (**A**) Time-kill curves for bacteria 1× MIC cefoxitin to check the inhibition kinetics of SA-GC and SA-PR. At the specific time intervals of post-treatment, viable cells were enumerated using the plate count method. (**B**) Time-kill curves for bacteria 1× MIC cefoxitin, oxacillin, and ciprofloxacin to check the inhibition kinetics of MRSA-GC and MRSA-PR. At the specific time intervals of post-treatment, viable cells were enumerated using the plate count method. (**C**) Representative crystal violet-stained test tubes showing biofilm formation in SA-GC, SA-PR, SA-GC + CIP, and SA-PR + CIP. Quantitative analysis of biofilm formation in SA-GC, SA-PR, SA-GC + CIP, and SA-PR + CIP represented as a percentage. (**D**) Representative crystal violet-stained test tubes showing biofilm formation in MRSA-GC, MRSA-PR, MRSA-GC + CIP, and MRSA-PR + CIP. Quantitative analysis of biofilm formation in MRSA-GC, MRSA-PR, MRSA-GC + CIP, and MRSA-PR + CIP represented as a percentage. (**E**) Spontaneous mutation frequency in the presence of PPEF (2×, 5×, 10× MIC) or without PPEF for 48 h. (**F**) Relative expression of virulence-associated genes (*fnbA, hla, spa, icaA, and coa*) in SA-PR and MRSA-PR. Data are shown as log_2_-fold change compared to respective controls.

Time-kill curve analyses (42) were conducted by culturing SA-GC, SA-PR, MRSA-GC, and MRSA-PR in the presence of cefoxitin at 1× MIC (0.5 µg/mL). In the absence of cefoxitin, bacterial growth reached approximately 10 log_10_ CFU/mL at 12 h for both SA-PR and MRSA-PR. The mean bacterial loads (log_10_ CFU/mL) over 12 h for cefoxitin-treated SA-PR and MRSA-PR are shown in Fig. 6A and B. The time-kill kinetics profile of SA-PR and MRSA-PR demonstrated a 3× log_10_-fold reduction in colony-forming units (CFU) after 4 and 5 h, respectively, ultimately resulting in bacterial death (Fig. 6A and B). MRSA-PR exhibited greater sensitivity to ciprofloxacin and oxacillin compared to their growth control strains MRSA-GC with 2× log_10_-fold reduction in CFU after 3 h (Fig. 6B). The checkerboard dilution method was used to evaluate the *in vitro* effects of PPEF in combination with CIP. Among the tested combinations, an indifferent effect was observed against all the evolved bacterial strains (see Table S4 at https://github.com/Vibhatandon1860/Microbiology-Spectrum). The MIC values of PPEF and CIP remained unchanged, whether tested individually or in combination. The fractional inhibitory concentration (FIC) index values were 1.5, 2, and 3 for SA-GC, SA-PR, and MRSA-PR, respectively. These findings indicate that PPEF does not exhibit synergy with ciprofloxacin in the evolved strains. The impact of PPEF on the emergence of spontaneous mutations was also examined in evolved strains. This study was based on the mutant selection window hypothesis, which suggests that an antimicrobial concentration range exists where drug-resistant mutants arise between the MIC of the evolved strains and the mutant prevention concentration (43). The effect of CIP on biofilm formation was evaluated using crystal violet staining, which showed significant inhibition of biofilm formation in SA-GC, SA-PR, MRSA-GC, and MRSA-PR, with reductions of 56.80%, 91.21%, 54.17%, and 90.89%, respectively (Fig. 6C and D) (44, 45). To induce such spontaneous mutations, bacterial cells were exposed to PPEF at concentrations exceeding the MIC value, 2×, 5×, and 10×. The objective was to assess the tolerance and spontaneous resistance development of these bacterial strains against PPEF. No colonies were observed beyond 1× MIC of PPEF treatment for SA-GC and MRSA-GC, suggesting that the frequency of spontaneous mutant generation is even lower than 1 in 10¹⁰ CFU for PPEF. The absence of colonies at 2×, 5×, and 10× MIC of PPEF indicates that the mutation selection window is narrow (Fig. 6E). The spontaneous mutation frequency at 2×, 5×, and 10× MIC for SA-PR and MRSA-PR was 0.011, 0.009, and 0 and 0.814, 0.720, and 0.376, respectively. This suggests that the evolved strains exhibit a high level of adaptability at elevated MICs once they have developed resistance to PPEF. Quantitative reverse transcriptase PCR (qRT-PCR) revealed that fnbA, hla, and spa were downregulated in SA-PR, with fold changes of 0.14, 0.07, and 0.32, respectively. A similar pattern of gene regulation was observed in MRSA-PR (Fig. 6F). The reduced expression of fnbA, hla, and spa likely impairs *S. aureus* ability to adhere to host tissues, evade immune responses, and cause tissue damage, thereby diminishing its virulence (46). These findings suggest that evolutionary pressure exerted by PPEF may have increased the susceptibility of evolved strains to other antibiotics. The enhanced sensitivity of the evolved strains could be attributed to adaptive responses under PPEF-induced selective pressure, potentially compromising resistance mechanisms or altering biofilm-associated pathways. Furthermore, the modulation of virulence gene expression in SA-PR and MRSA-PR aligns with their increased antibiotic susceptibility. The observed differences between evolved and control strains underscore the potential of exploiting evolutionary adaptations to improve antibiotic efficacy.

### Molecular detection of resistance genes

**Fig 7:**
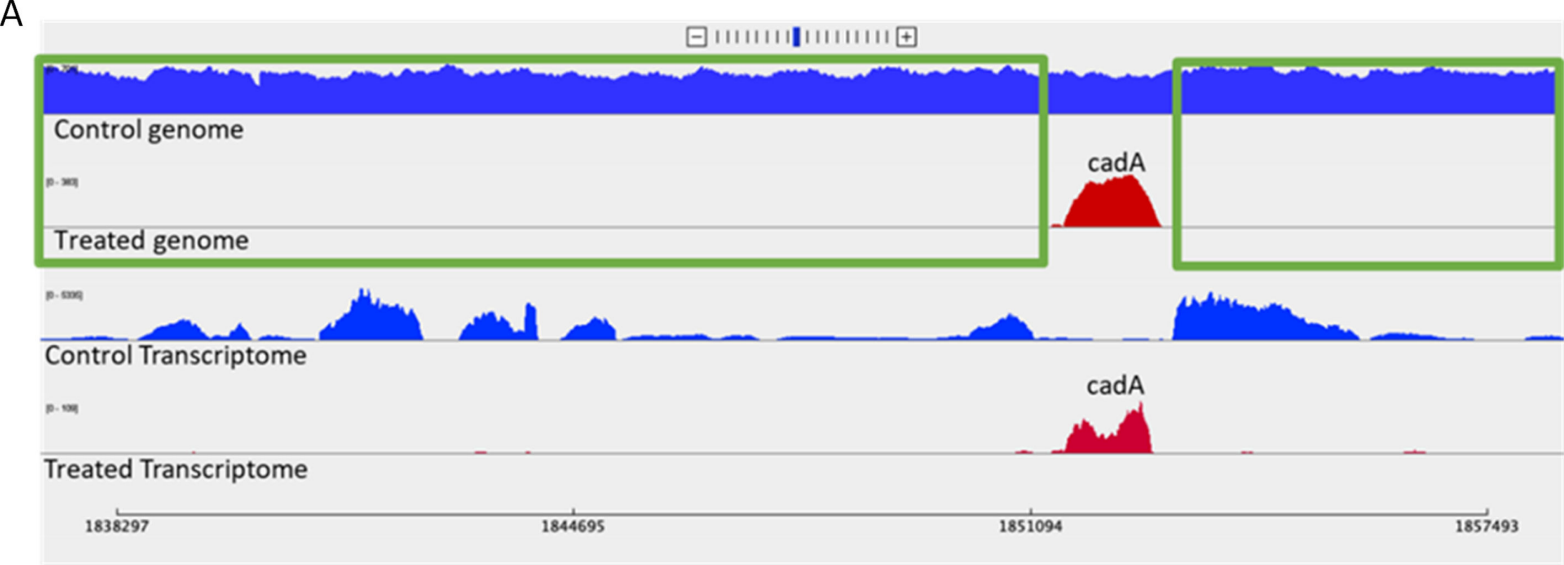
Comparison of sashimi plots of treated and control A. Sashimi plot for the molecular identification of the genomic coordinates in MRSA-GC versus MRSA-PR.

The deletion of the ~20 kb region in the MRSA-PR genome was observed through genomics analysis/Sashimi plot (47). Serial passages may influence bacterial adaptation through genotypic and phenotypic alterations. In a closely related organism, genome alterations (i.e., gene insertions, deletions, and repetitive sequences) were observed during *in vitro* passage. The omitted region spanning from bp 1838296 to bp 1857494 bp was further confirmed by no reads aligning in the treated transcriptome, but the “*cadA*” gene was preserved at genomic coordinate 1851094 (Fig. 7). This signifies the role of the *cadA* gene involved in PPEF resistance. It has already been shown that hospital-acquired MRSA can lose its antibiotic resistance genes when exposed to lower antibiotic selective pressure in the community (48). This finding implicates the significance of the *cadA* gene in drug metabolism leading to the survival of MRSA-PR in the presence of the PPEF molecule.

### Validation of transcriptional gene expression by quantitative real-time PCR (qPCR)

**Fig 8:**
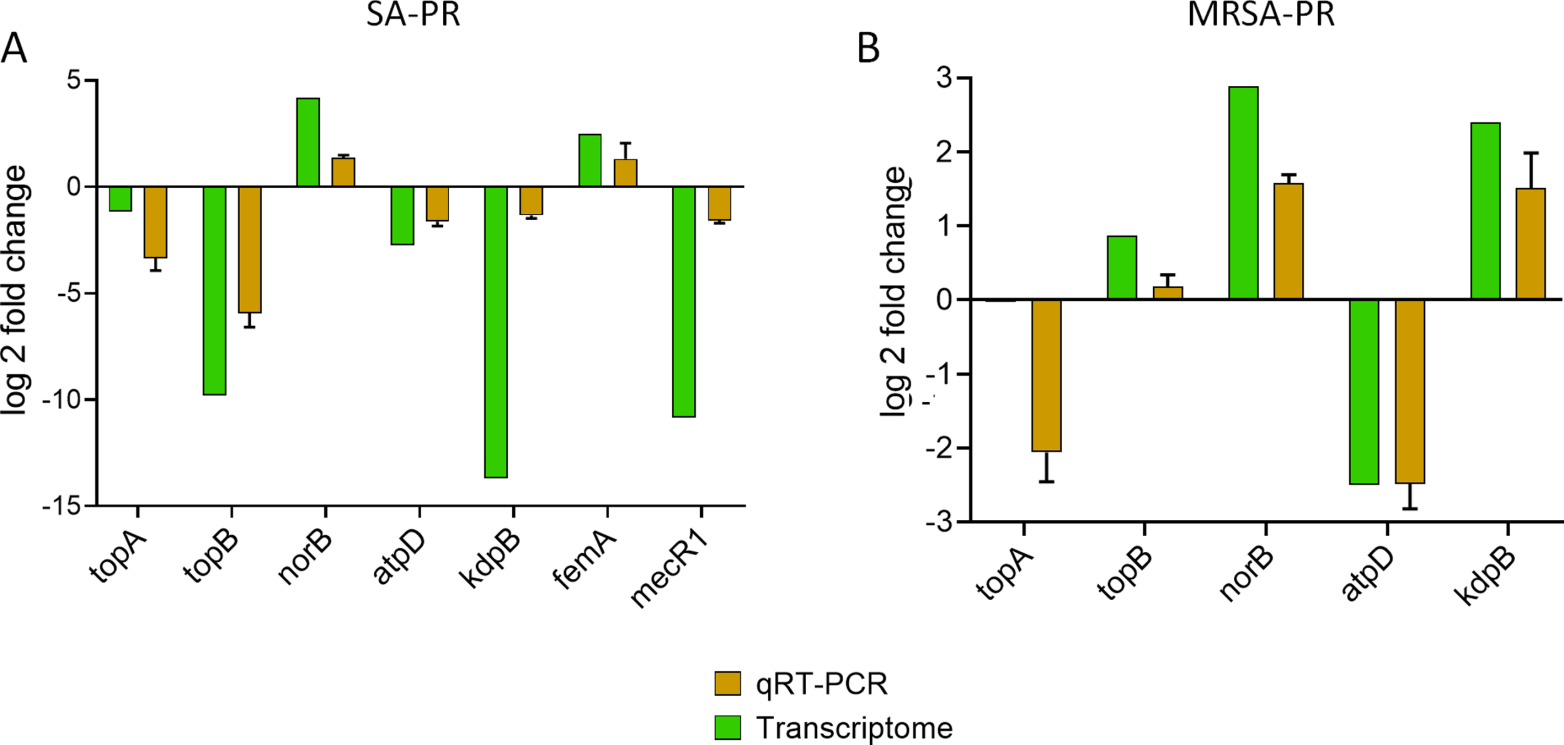
The fold change of the gene determined through NGS and validated by real-time PCR. (A) Expression profile of *topA, topB, norB, atpD,* and *kdpB* in SA-PR. (B) Expression profile of *topA, topB, norB, femA, mecR1, atpD,* and *kdpB* in MRSA-PR. Results are presented in triplicate of mean ± SD.

The findings achieved in qPCR validated our RNA seq data. In SA-PR, the log_2_-fold changes for *atpD, topB*, and *topA* were −2.48, 0.183, and −2.06, respectively. Similar changes were also observed in the transcriptomics data (Fig. 8A). In MRSA-PR, *atpD* was downregulated with a log_2_-fold change of −1.63, while *topA* and *topB* were downregulated with log_2_-fold changes of −3.35 and −5.93, respectively. Additionally, *femA* and *norB* genes were significantly upregulated, with log_2_-fold changes of 1.31 and 1.36, respectively, under PPEF treatment, as shown in real-time PCR (Fig. 8B). In general, there was a positive correlation between the two methods. However, the *kdpB* and *MecR1* genes were altered to a greater degree in next generation sequencing (NGS) compared with real-time qPCR, indicating that NGS analysis may be more sensitive to changes than real-time qPCR.

### Limitations

Despite its valuable contributions, this study has limitations. The experiments were conducted in controlled settings, requiring validation in animal models and human infections. While MRSA undergoes genetic deletions that enhance antibiotic sensitivity, the stability of these adaptations across different environments remains uncertain. Additionally, PPEF exposure increases susceptibility to other antibiotics, but prolonged use may trigger compensatory mechanisms that restore or alter resistance.

### Conclusion

**Fig 9:**
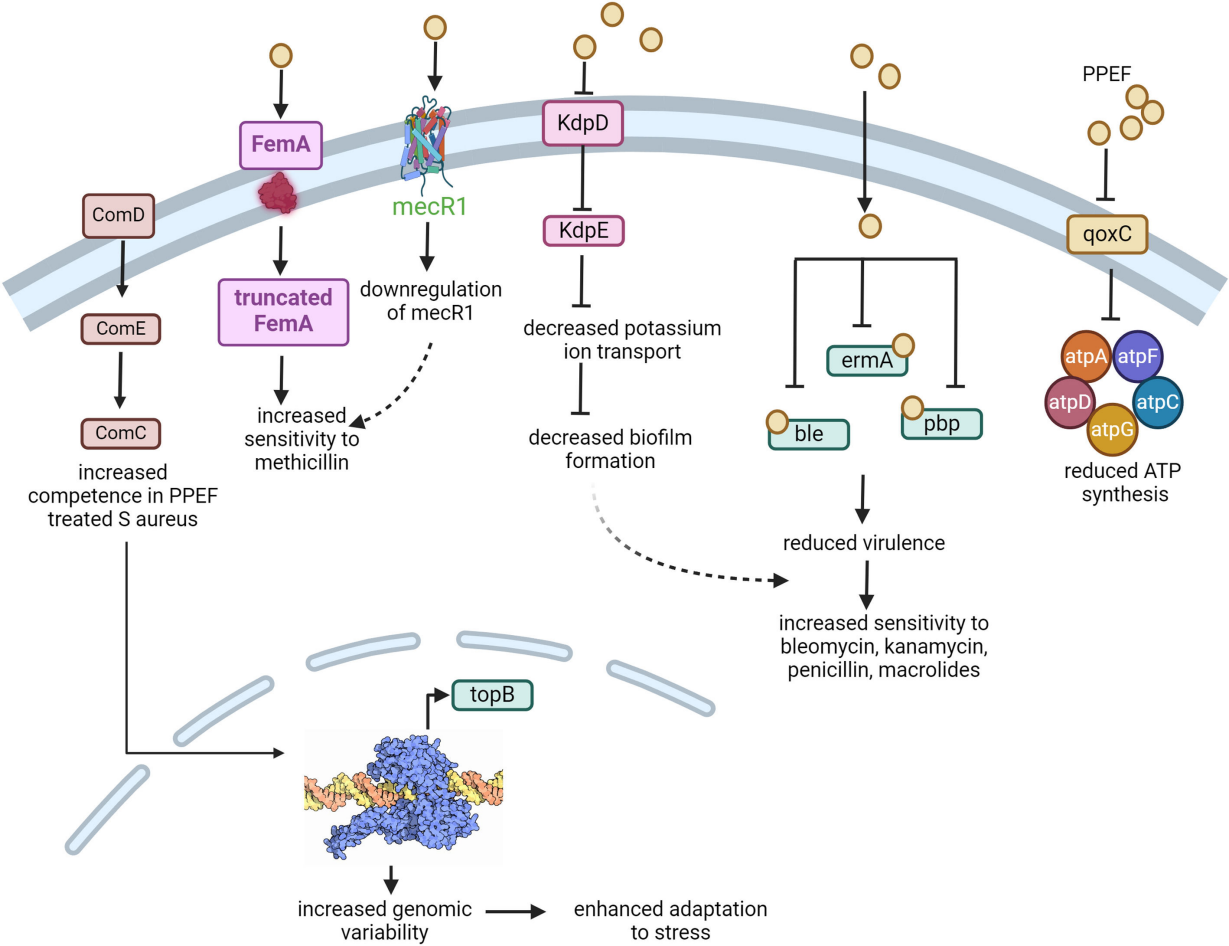
Proposed model for the evolved *S. aureus* against PPEF. Regulation of the spectrum of genes involved in two-component system, replication, and pathogenesis in *S. aureus* in the presence of PPEF for its adaptive evolution to known antibiotics.

A correlation could be established that alterations in the expression of genes are associated with the transcriptomic rewiring that may mediate the resistant phenotypes in PPEF-evolved *S. aureus*. Resistance to PPEF and enhanced sensitivity to known antibiotics, namely kanamycin, bleomycin, macrolides, and penicillin, is associated with the upregulation of genes of the *comGC* system and downregulation of *topB,* which increases the genomic variability for its better adaptation. In contrast, downregulation of *kdpE, mecR1, ble, pbp,* and *qoxC* genes results in reduced virulence. This study provided insights into the regulatory mechanism for the acquisition of resistance to PPEF and susceptibility to known antibiotics (Fig. 9). Real-time PCR also confirmed the contribution of genes, namely *femA, atpD, topB,* and *topA,* for the development of the resistance to PPEF and increased sensitivity to known antibiotics. Although the exact mechanisms of PPEF resistance for *S. aureus* are not yet fully understood, our research suggests that type IA topoisomerase is a potential antibacterial drug target, and topoisomerase inhibitors have great potential to be a new and safe class of antibacterial agents for treating MRSA and VRSA. Overall, our outputs contribute to clarifying the placement of PPEF as an alternative candidate molecule against *S. aureus*.

## MATERIALS AND METHODS

### Bacterial strains and culture conditions

*Staphylococcus* strains (ATCC 25923 and ATCC 43300) were collected from the American Type Culture Collection (ATCC). ATCC 25923 is a clinical isolate, designated as Seattle 1945, and used as a standard control strain under laboratory conditions. ATCC 43300 is a reference strain of MRSA. It is used as a quality control strain to test the methicillin susceptibility of clinical isolates. For routine cultures, *Staphylococcus* was grown in Mueller-Hinton broth (MHB). Cultures were incubated at 37°C either with shaking (230 rpm) or under static conditions.

### Antibacterial susceptibility test

MIC was determined by broth microdilution method according to CLSI methods (18). The bacterial suspensions of 1.0 × 10^6^ CFU were seeded in 96-well plates. The experiment included growth and sterile controls: one with bacterial inoculum without antibiotics and another with broth devoid of both bacteria and antibiotics. Furthermore, antibiotics were added at increasing concentrations ranging from 0.25 to 128 µg/mL, including vancomycin, ciprofloxacin, kanamycin, gentamicin, co-trimoxazole, colistin, tetracycline, chloramphenicol, ofloxacin, ampicillin, oxacillin, cefoxitin, cefotaxime, and imipenem, followed by incubation at 37°C for 24 h. MIC values were scored as the concentration at which no visible growth of bacterium was observed and detected the OD_600_ by Tecan Microplate Reader (infinite_M200 PRO_). The absolute MIC value was scored as the minimum concentration that inhibited growth by 90% at 24 h. This experiment was done in triplicates.

### Generation of PPEF resistance in *S. aureus*

*S. aureus* ATCC 25923 and ATCC 43300, a fully susceptible strain, were cultured in MHB that contained sub-MIC values of PPEE, which is a previously published procedure (16). *S. aureus* ATCC 25923 and ATCC 43300 cells were grown to exponential phase and diluted to OD_600_ of 0.01 in 1 mL of MHB with PPEF. One percent of the population was transferred to every 24 h of interval in the fresh media with 0.25 µg/mL of PPEF. Briefly, *S. aureus* strains were grown overnight from a single colony. Approximately 10^6^ CFUs were challenged initially with a sub-lethal dose (1/2× MIC) of PPEF and grown for 24 h. After every 24 h (approximately eight generations), *S. aureus* cells were passaged serially through 100-fold dilution in a batch of 1 mL culture with gradual increase in concentration of PPEF in MHB. After ~440 generations (55 days), the obtained *S. aureus* population was resistant to PPEF. Experiments were performed with three biological replicates (49).

### Visual pigment loss in the evolved *S. aureus*

The evolved strains of *S. aureus* were incubated in MHB at 37°C for 14 h, followed by a dilution of 1:100 in 5 mL MHB, grown until OD_600_ of 1.0. The culture was then centrifuged and washed with 1× phosphate-buffered saline (PBS) to remove all traces of media. Similarly, cultures were also harvested at different days. Subsequently, the cells were centrifuged in a 50 mL tube, and the supernatant was discarded. The pellet was then observed for any phenotypic changes (21).

### Bacterial growth curves

To check the bacterial growth response of evolved strains, overnight cultures of *S. aureus* 25923, SA-GC, SA-PR, MRSA 43300, MRSA-GC, and MRSA-PR were diluted 1:100 and incubated for 3 to 4 h at 37°C. The secondary cultures were then further diluted in 5 mL falcon tubes to a concentration of ~10^5^ CFU/mL in MHB. These cultures were incubated at 37°C for 20 h. OD_600_ was taken every 30 min until 10 h of growth from the 11th to the 20th h OD were taken every 1 h of interval (50). Doubling times were determined using the GrowthRates 6.2.1 software.

### Library preparation and next-generation sequencing of evolved *S. aureus* strains

#### Genomic

DNA extraction was carried out using the QIAGEN Genomic DNA Purification Kit (Sigma-Aldrich, USA). Library preparation was performed using the Nextera XT DNA library preparation protocol. A total of 1 ng of Qubit-quantified genomic DNA was fragmented and adaptor tagged using Amplicon Tagment Mix provided in the Nextera XT Kit. The adapter sequence used was 5′-AGATCGGAAGAGCGTCGTGTAGGGAAAGAGTGTAGATCTCGGTGGTCGCCGTATCATT-3′, and index adapter 5′-GATCGGAAGAGCACACGTCTGAACTCCAGTCAC GGATGACTATCTCGTATGCCGTCTTCTGCTTG-3′-tagged DNA was subjected to 12 cycles of indexing-PCR (72°C for 3 min followed by denaturation at 95°C for 30 s, cycling at 95°C for 10 s, 55°C for 30 s, 72°C for 30 s, and 72°C for 5 min) to enrich the adapter-tagged fragments. The PCR product was purified using HighPrep Beads (Magbio). Quantification and size distribution of the prepared library were determined using Qubit fluorimeter and Agilent TapeStation, respectively, according to the manufacturer’s instructions.

Libraries were sequenced using Illumina NovaSeq 6000. Also, 1–2 GB reads were generated per sample with the coverage of ~200×. The genome reads were assembled by Unicycler (51), and annotation of the generated genome was done using RAST and Prokka (52). Single-nucleotide change analysis in the PPEF-evolved strains compared to control strains was done by Snippy software (53).

#### Transcriptome

RNA extraction was carried out using the QIAGEN RNA Purification Kit (Sigma-Aldrich, USA). RNA sequencing libraries were prepared with the Illumina-compatible NEBNext Ultra Directional RNA Library Prep Kit (New England BioLabs, MA, USA). A total of 2.5 µg of total RNA was taken for rRNA depletion using the Ribo-Zero rRNA Removal Kit (Bacteria). Approximately 30 ng of Qubit-quantified ribo-depleted RNA was taken for fragmentation and priming. The fragmented and primed mRNA was further subjected to first strand synthesis in the presence of actinomycin D (Gibco, Life Technologies, CA, USA) followed by second strand synthesis. The double-stranded cDNA was purified using HighPrep PCR magnetic beads (Magbio Genomics Inc, USA). The purified cDNA was end-repaired, adenylated, and ligated to Illumina multiplex barcode adapters as per NEBNext Ultra Directional RNA Library Prep Kit protocol. The adapter-ligated cDNA was purified using HighPrep beads and was subjected to 15 cycles of indexing-PCR (37°C for 15 min followed by denaturation at 98°C for 30 s, cycling [98°C for 10 s, 65°C for 75 s], and 65°C for 5 min) to enrich the adapter-ligated fragments. The final PCR product (sequencing library) was purified with HighPrep beads, followed by library quality control check. The Illumina-compatible sequencing library was initially quantified by Qubit fluorimeter (Thermo Fisher Scientific, MA, USA), and its fragment size distribution was analyzed on Agilent TapeStation. Libraries were sequenced using Illumina NovaSeq 6000 paired-end sequencing with 150 × 2 bp chemistry.

#### Transcriptomic analysis

Prokseq pipeline was used for the analysis. The tool is fully automated and has its own default parameters. First, the FASTQ file of the treated as well as control samples (treated and control) was checked for the quality of the reads by using filters in Babraham Bioinformatics-FastQC: a quality control analysis tool. Furthermore, the reads were mapped with the reference genome using bowtie2 to deduce the information for the conserved sequences. The sequencing depth and coverage were also calculated. Feature counts were used for the total number of reads aligned per gene. Normalized gene expression was calculated in the form of transcripts per million and counts per million. DESeq2, edgeR, and NOISeq were used to analyze the differential expression of genes (54).

### Gene enrichment analysis

For functional annotation of GO and analysis of KEGG pathway enrichment, we used the web-based DAVID v.6.8 tool. DAVID is a significant source for functional evaluation of the high-throughput gene expression profiles. The upregulated and downregulated gene list obtained after the transcriptomics analysis was taken as the primary list and submitted to DAVID. The results from DAVID were further imported to GO plot in R Studio. The GO Bubble was used to visualize the function enrichment of the top DEGs, which facilitated the combination and integration of expression data results from RNA seq analysis. DAVID syndicates KEGG pathways and GO, which delivers a fundamentally organized pathway network or GO from the DEG data set. Also, the study of molecular/biological function GO and enrichment of pathways analysis were conducted for DEGs, and *P*-values <0.01 were considered to be significant (55).

### PPI network analysis

The PPI analysis was conducted on all the common DEGs using the STRING30 database (version 12.0, http://www.string-db.org/) with the highest confidence parameter set to 0.9. In brief, a list of proteins was provided as input, allowing STRING to search for their neighboring interactors, proteins that directly interact with the input proteins. STRING then generated the PPI network, encompassing all these proteins and their interactions. These interactions were derived from high-throughput lab experiments and existing knowledge in curated databases, ensuring a high level of confidence (sources: experiments, databases; score ≥0.90) (56).

### Time-kill assay

To evaluate the effect of cefoxitin, oxacillin, and ciprofloxacin on bacterial growth, a time-response growth curve was prepared based on the NCCLS standards (57). The 1 mL bacterial suspensions, with a cell density of 10⁷ CFU/mL, were treated with 1× MIC concentrations of oxacillin (0.25 µg/mL), ciprofloxacin (0.25 µg/mL), and cefoxitin (1.0 µg/mL for SA-PR and 2.0 µg/mL for MRSA-PR), respectively. In the control tube, an equivalent volume of sterile Milli-Q water was added. In the control tube, an equal volume of sterile Mili-Q water was added. These cultures were incubated at 37°C with constant stirring at 150 rpm. Broth aliquots were collected at different time points (0, 1, 2, 3, 4, 5, 6, 8, 10, 12, and 24 h), serially diluted in saline solution, plated on Müller-Hinton agar media, and grown for 24 h at 37°C to determine the total CFUs in each culture at respective time points. Bactericidal activity is defined as a greater than 3 log_10_-fold decrease in colony-forming units (surviving bacteria), which is equivalent to 99.9% killing of the inoculum.

### Checkerboard assay

The Synergistic effect of the combination of PPEF with ciprofloxacin was determined by the checkerboard method adapted from a previously described method (43). As per the method, the antibacterial activity of the combination of PPEF and ciprofloxacin was assessed against bacterial strains by microdilution assays employing double antimicrobial gradient. In the present study, the combinations chosen were as follows: 1/2× MIC of CIP fixed and twofold serial dilutions of PPEF (64 μg/mL to 0.125 μg/mL) were used. Similarly, 1/2× MIC of PPEF fixed and twofold serial CIP (64 μg/mL to 0.125 μg/mL) were used. Minimum inhibitory concentration was determined using Tecan Microplate Reader at 600 nm. The effect of combination of drug, defined as per the FIC index, was calculated as follows: ΣFIC = FIC A + FIC B, where FIC A = (MIC of PPEF in combination with 1/2× MIC of ciprofloxacin) and FIC B = (MIC of ciprofloxacin in combination with 1/2× MIC of PPEF).

A result of FIC = 4.0 was considered antagonistic, FIC = 1 to 4 indifferent, FIC = 0.5 to 1 additive, and FIC < 0.5 synergistic.

### Determination of spontaneous mutation frequency to resistance

The evolved *S. aureus* strain (SA-GC, SA-PR, MRSA-GC, MRSA-PR) cells were grown overnight, then diluted to fresh media to a final concentration of 10^10^ to 10^11^ CFU/mL. An aliquot (100 µL) of bacterial suspension was spread onto plates of cation-adjusted Mueller-Hinton agar containing the PPEF at 2×, 5×, and 10× dilution of MIC. The plates were incubated aerobically at 37°C for 48 h. Resistance studies were defined as a MIC ≥4 times higher than that of the parent. The spontaneous mutation frequency was determined from the fraction of colony number observed in the presence of PPEF to the colony number observed without treatment after 48 h (16).

### Antibiofilm assay

The biofilm assay was conducted in a 1 mL glass tube. Firstly, the SA-GC, SA-PR, MRSA-GC, and MRSA-PR suspension at 10^6^ cfu/mL was prepared and dispensed into each well in the 1 mL glass tube. For the dose-dependent antibiofilm assay, CIP was tested at a concentration of 0.125 µg/mL. Uninoculated MHB was also included as the blank control. The tubes were incubated at 37°C for 24 h. After incubation, the tubes were washed three times with 1× PBS to eliminate non-adherent bacteria and fixed with 99% (vol/vol) methanol for 15 min. The wells were allowed to dry in a laminar flow. The attached biofilm cells were stained using filtered 0.5% crystal violet for 15 min at room temperature. The excess stain was removed by rinsing with water, and crystal violet-bound cells were solubilized with 70% ethanol. The released stain was measured at 570 nm using a microplate reader (Tecan). Analysis was performed on three independent occasions and three technical replicates for each (17).

### qRT-PCR

For the experiment, evolved *S. aureus* strains SA-PR and MRSA-PR were grown with PPEF, while SA-GC and MRSA-GC strains were grown in the absence of PPEF. All cultures were taken out when an OD_600_ of 0.6 to 0.7 or approximately 10^8^ cells was reached. Total RNA was isolated and dissolved in RNase-free water, followed by quantification using a Nanodrop spectrophotometer. The integrity of the RNA was checked by running a 2% formaldehyde agarose gel. The isolated RNA was further used for cDNA synthesis using the high-capacity cDNA reverse transcription kit. For the template, 1 µg of RNA was reverse-transcribed into cDNA at 25°C for 10 min, 37°C for 120 min, and 85°C for 5 min. The qRT-PCRs were conducted with a total reaction volume of 10 µL containing 20 ng of cDNA, 10 µM of forward and reverse primers, 5 µL of SYBR Green mixture (PowerUp SYBR Green Master Mix of Applied Biosystems), and 2 µL of nuclease-free water. Amplification was performed in triplicate, starting with initial denaturation followed by 40 cycles of denaturation, annealing, and extension. For data analysis, relative gene expression was normalized to U16s, a housekeeping gene with constitutive expression. The primers used in this study are detailed in Table S1 at https://github.com/Vibhatandon1860/Microbiology-Spectrum (17).

### Statistical analysis

Experimental/laboratory evolution of *S. aureus* strains was performed in triplicate with three independent experiments up to 440 generations. Transcriptomics of each sample was performed in duplicate and genomics with coverage of more than 200×. MIC_90_ and resistant/sensitivity experiments against 14 antibiotics were performed thrice in triplicate. The experimental data are presented as mean ± standard deviation from three independent experiments, unless specified otherwise. Statistical significance was calculated using GraphPad Prism 8. Data analysis involved two-way analysis of variance. *P*-values of (*P < 0.01, ** <0.001, ****P < 0.0001) were considered statistically significant, and ns was considered not significant.

## Data Availability

The NGS data have been deposited in NCBI’s Sequence Read Archive (SRA) and are accessible through BioProject PRJNA1078546, PRJNA1078742, PRJNA1079528, and PRJNA1082042. All other data associated with this study are present in the article and supplemental material.
